# MicroRNA-130b improves renal tubulointerstitial fibrosis via repression of Snail-induced epithelial-mesenchymal transition in diabetic nephropathy

**DOI:** 10.1038/srep20475

**Published:** 2016-02-03

**Authors:** Xiaoyan Bai, Jian Geng, Zhanmei Zhou, Jianwei Tian, Xiao Li

**Affiliations:** 1 Division of Nephrology, Nanfang Hospital, Southern Medical University, National Clinical Research Center for Kidney Disease, State Key Laboratory of Organ Failure Research, Guangdong Provincial Institute of Nephrology, Guangzhou, 510515, China; 2 Department of Pathology, Nanfang Hospital, Southern Medical University, Guangzhou, 510515, China; 3 Department of Emergency, Nanfang Hospital, Southern Medical University, Guangzhou, 510515, China

## Abstract

MicroRNA-130b (miR-130b) downregulation has been identified in diabetes, but the role and mechanisms for miR-130b in mediating renal tubulointerstitial fibrosis in diabetic nephropathy (DN) remain unknown. We demonstrated that plasma miR-130b downregulation exhibited clinical and biological relevance as it was linked to increased serum creatinine, β2-microglobulin and proteinuria, increased Snail expression and tubulointerstitial fibrosis in renal biopsies of DN patients. MiR-130b inhibitor caused *Snail* upregulation and enhanced molecular features of epithelial-to-mesenchymal transition (EMT) in high glucose (30 mM) cultured NRK-52E cells. In contrast, miR-130b mimic downregulated *Snail* expression and increased epithelial hallmarks. Notably, *Snail* was identified as an miR-130b direct target and inversely correlated with E-CADHERIN expression. Furthermore, the miR-130b-dependent effects were due to *Snail* suppression that in turn deregulated E-CADHERIN, VIMENTIN, COLLAGEN IV and α-smooth muscle actin (α-SMA), key mediators of EMT. These effects were reproduced in streptozotocin-induced diabetic rats. Thus, we propose a novel role of the miR-130b-SNAIL axis in fostering EMT and progression toward increased tubulointerstitial fibrosis in DN. Detection of plasma miR-130b and its association with SNAIL can be extrapolated to quantifying the severity of renal tubulointerstitial fibrosis. Targeting miR-130b could be evaluated as a potential therapeutic approach for DN.

The incidence and prevalence of diabetes are rapidly rising worldwide. About 10% of patients with diabetes develop diabetic nephropathy (DN) and up to 40% of diabetic patients are affected by renal failure, and thus is the leading cause of end-stage renal disease (ESRD)^1^. In China, diabetes has become a major public health problem with the incidence of type 2 diabetes rising to 9%^2^. Tight glycemic control and inhibition of the rennin-angiotensin system (RAS) have been proven to reduce the incidence and slow the progression of diabetic nephropathy^3^. However, the prevalence of DN still remains relatively high, and many patients on RAS inhibitors still progress to ESRD. Therefore, identifying valuable biomarkers is of great significance for early diagnosis and treatment of the disease.

In diabetes, tubules are vulnerable to injuries and tubulointerstitial fibrosis has been recognized as a final common pathogenic process. Emerging lines of evidences suggest that reactivation or dysregulation of key developmental signaling play a critical role in the pathogenesis of chronic tissue destruction and progressive loss of kidney function^4^.

MicroRNAs (miRNAs) are highly conserved small non-coding RNAs involved in numerous biologic processes. MiRNAs recognize complementary sequences in the 3′-untranslated region (3′-UTR) of target mRNAs leading to decreased protein expression either by mRNA degradation and/or by translational repression^5^,^6^,^7^. MiR-130 has been linked to mesenchymal differentiation and hypoxic response modulation in tumor angiogenesis^8^. Moreover, varied levels of miR-130b have been documented in several kinds of diseases, with increased expression in tissues of melanoma^9^ and colorectal cancer^10^, but decreased in the serum of patients with type 2 diabetes^11^, tissues of endometrial cancer^12^ and pituitary adenomas^13^. However, whether miR-130b regulates renal tubulointerstitial fibrosis in diabetic nephropathy and the underlying mechanisms have not been elucidated.

Snail, the fundamental member of the family of Snail transcriptional factors, has emerged as the most established master regulator of epithelial-mesenchymal transitions (EMT)^14^. Several miRNAs have been shown to modulate the activity of Snail. It has been reported that miR-133 promotes cardiac reprogramming by directly repressing Snail^15^. MiR-29b downregulates Snail in colorectal cancer cells^16^ and miR-30a negatively regulates *Snai1*-mediated EMT during peritoneal fibrosis^17^. Furthermore, miR-125b functions as a key mediator for Snail-induced stem cell propagation and chemoresistance^18^. However, whether miR-130b modulates the activity of Snail in diabetic nephropathy has not been well studied.

In this study, we found that plasma miR-130b downregulation associated with increased serum creatinine and β2-microglobulin, increased Snail expression and increased tubulointerstitial fibrosis in DN. MiR-130b inhibition could induce Snail signaling activation and enhance EMT *in vitro* and *in vivo*.

## Results

### Plasma miR-130b downregulation contributes to increased tubulointerstitial fibrosis and unfavorable renal function in DN patients

**Figure 1:**
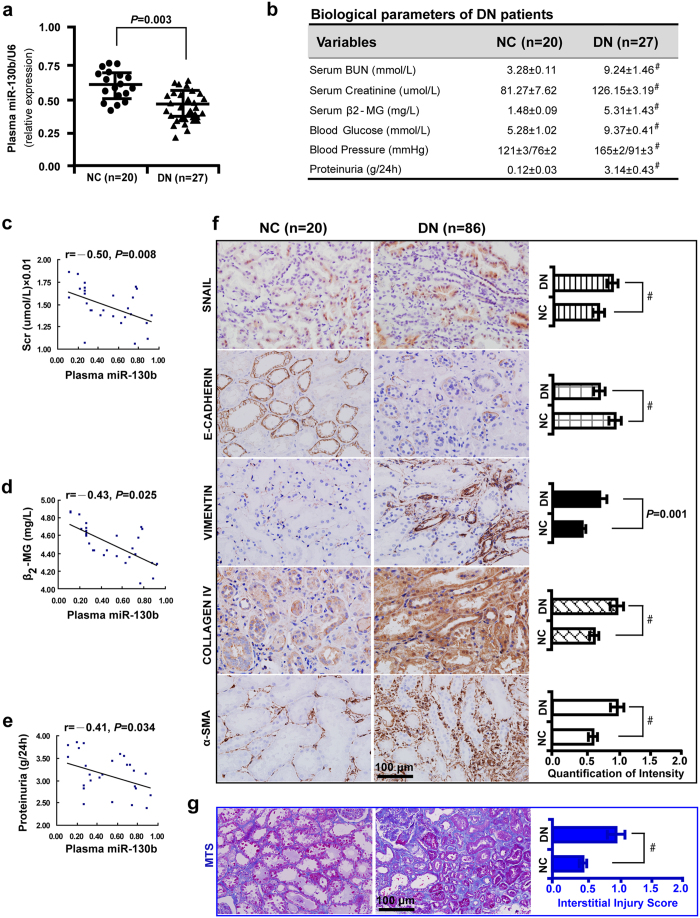
Plasma miR-130b downregulation contributes to increased tubulointerstitial fibrosis and unfavorable renal function in diabetic nephropathy (DN) patients. (**a**) Decreased plasma miR-130b in 27 DN patients compared with 20 healthy controls. (**b**) Increased BUN, serum creatinine, β2-microglobulin, blood glucose, blood pressure and urine albumin in 27 DN patients compared with 20 normal controls using Mann-Whitney U-test. (**c**) Pearson correlation analysis demonstrated the inverse correlations of plasma miR-130b with serum creatinine, (d) β2-microglobulin and (**e**) proteinuria in 27 DN patients, respectively. (**f**) Upregulation of SNAIL, VIMENTIN, COLLAGEN IV and α-SMA but downregulation of E-CADHERIN by immunohistochemistry and quantification of the staining intensity in 86 renal biopsy samples compared with 20 normal controls. (**g**) Increased renal tubulointerstitial fibrosis in DN patients by MTS and the quantification analysis. Results are presented as mean ± SD of three independent experiments. ^#^*P* *<* 0.0001. NC, normal control; DN, diabetic nephropathy; MTS, Masson’ s trichrome stain.

**Figure 2:**
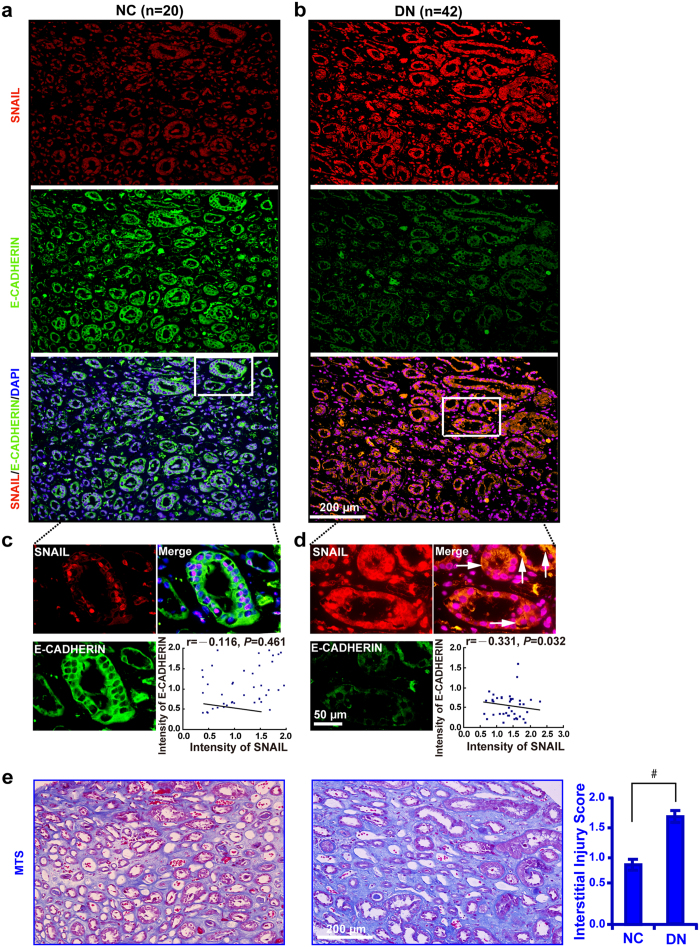
SNAIL negatively correlates with E-CADHERIN and corresponded with increased renal tubulointerstitial fibrosis in DN. (**a**) Immunofluorescence staining of SNAIL and E-CADHERIN in renal tubules of 20 normal controls and (**b**) 42 snap-frozen DN samples. (**c**) Magnified images of the boxed area showing increased E-CADHERIN expression. (**d**) Yellow color represents the co-localization between SNAIL and E-CADHERIN (arrows). (**e**) Increased renal tubulointerstitial fibrosis and increased interstitial injury score in DN compared with NC by MTS. Results are presented as mean ± SD of three independent experiments. ^#^*P* *<* 0.0001. NC, normal control; DN, diabetic nephropathy; MTS, Masson’ s trichrome stain.

Compared with 20 normal controls, plasma miR-130b mRNA level was considerably decreased in 27 DN patients (Fig. 1a). The level of BUN, serum creatinine, β2-microglobulin, blood glucose, blood pressure and proteinuria were increased in the DN patients compared with normal controls (Fig. 1b). Inverse correlations between plasma miR-130b with serum creatinine (Fig. 1c), β2-microglobulin (Fig. 1d), and proteinuria (Fig. 1e) were detected. Immunohistochemistry and quantification of the staining intensity (Fig. 1f) revealed that in 86 renal biopsies of DN, the level of SNAIL, VIMENTIN, COLLAGEN IV and α-SMA was upregulated with E-CADHERIN downregulation. The interstitial fibrosis was exacerbated in DN compared with normal controls as detected by MTS and the quantification analysis (Fig. 1g). Immunofluorescence co-labeling revealed no correlation between the expression of SNAIL and E-CADHERIN in normal control kidney tissues (Fig. 2a,c), but the correlation reached statistical significance in renal biopsies of diabetic patients (Fig. 2b,d) and corresponded with increased renal tubulointerstitial fibrosis (Fig. 2e). These results suggest that plasma miR-130b negatively correlates with serum creatinine, β2-microglobulin, and proteinuria in DN patients.

### MiR-130b ablation enhances *Snail*-induced EMT *in vitro*

**Figure 3:**
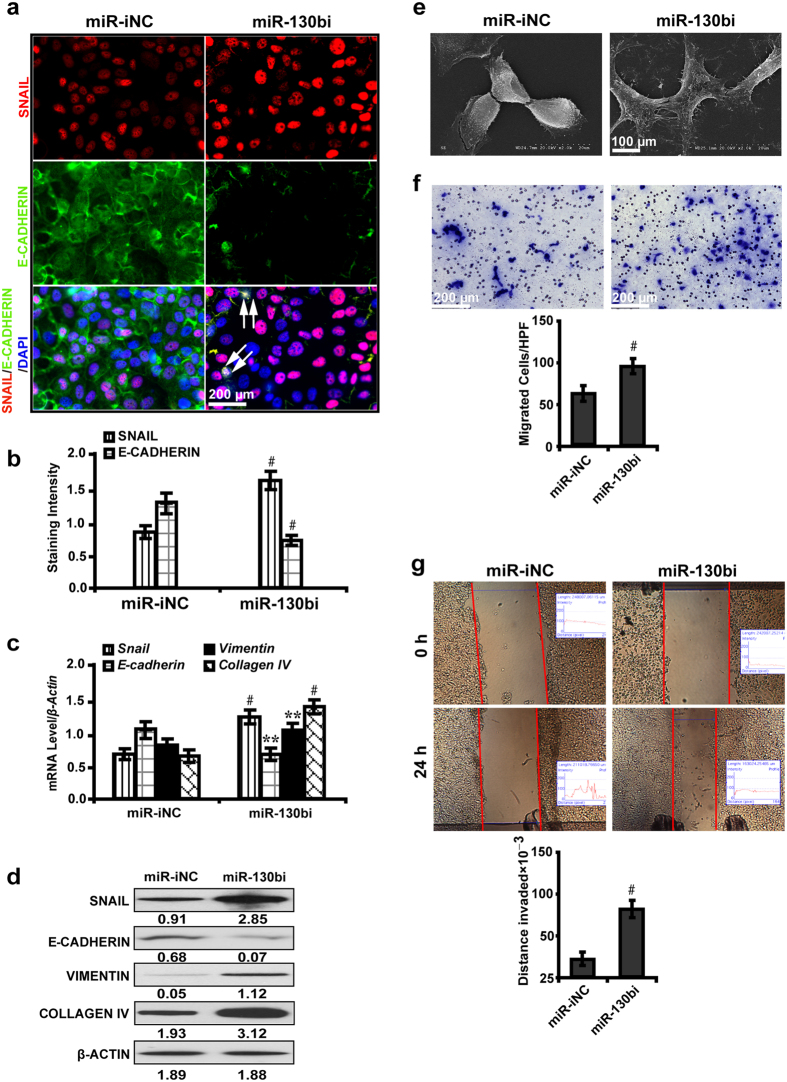
MiR-130b ablation enhances *Snail*-induced EMT *in vitro*. (**a**) MiR-130b inhibitor increased the expression of SNAIL and co-localized with E-CADHERIN (double arrows) (immunofluorescence). (**b**) Decreased E-CADHERIN by quantification of the staining intensity. (**c**) MiR-130b inhibitor upregulated the mRNA level of *Snail*, *Vimentin* and *Collagen IV*, but downregulated *E-cadherin* by qRT-PCR; miR-130b inhibitor increased SNAIL, VIMENTIN and COLLAGEN IV but decreased E-CADHERIN by (**d**) Western blot analysis. (**e**) MiR-130b inhibitor induced phenotypic changes of NRK-52E cells with elongated spindle-shaped cell bodies like fibroblasts by SEM. (**f**) Increased migrated cells treated with miR-130b inhibitor by transwell assay. (**g**) Longer invaded distances in NRK-52E cells treated with miR-130b inhibitor by wound healing assay. Results are presented as mean ± SD of three independent experiments. **P *<* 0.01; ^#^*P* *<* 0.0001. miR-iNC: miRNA inhibitor negative control; miR-130bi: miR-130b inhibitor; SEM, Scanning Electron Microscope.

Given the critical role of plasma miR-130b and its possible correlation with increased tubulointerstitial fibrosis in renal biopsies of DN patients (Fig. 1), we next investigated the potential effect of miR-130b inhibition on EMT and fibrosis-related molecules using an *in vitro* model system. NRK-52E cells were cultured in high glucose medium (30 mM) for 24 hours followed by treatment with miR-130b inhibitor for another 48 hours. As illustrated using immunofluorescence microscopy, miR-130b inhibition resulted in marked increase in the expression of SNAIL and co-localized with E-CADHERIN (Fig. 3a, double arrows), the level of which decreased (Fig. 3b). Quantitative real-time RT-PCR analysis showed that miR-130b inhibitor upregulated the mRNA level of *Snail*, *Vimentin* and *Collagen IV*, but downregulated *E-cadherin* (Fig. 3c). Western blot analysis revealed that miR-130b abrogation caused considerable increase in the expression of SNAIL, corresponded with increased VIMENTIN and COLLAGEN IV but decreased E-CADHERIN (Fig. 3d). MiR-130b abolishment instigated morphological changes of NKR-52E cells with elongated spindle-shaped cell bodies like fibroblasts, indicating a phenotypic transformation from epithelial to mesenchymal properties (Fig. 3e). Notably, miR-130b depletion enhanced the ability of NRK-52E cells to migrate (Fig. 3f) and invade (Fig. 3g) as detected by transwell and wound healing assay. These data suggest that activation of *Snail* signaling by miR-130b inhibitor promotes the expression of fibrosis-related genes and EMT process.

### MiR-130b mimic suppresses *Snail*-induced EMT *in vitro*

**Figure 4:**
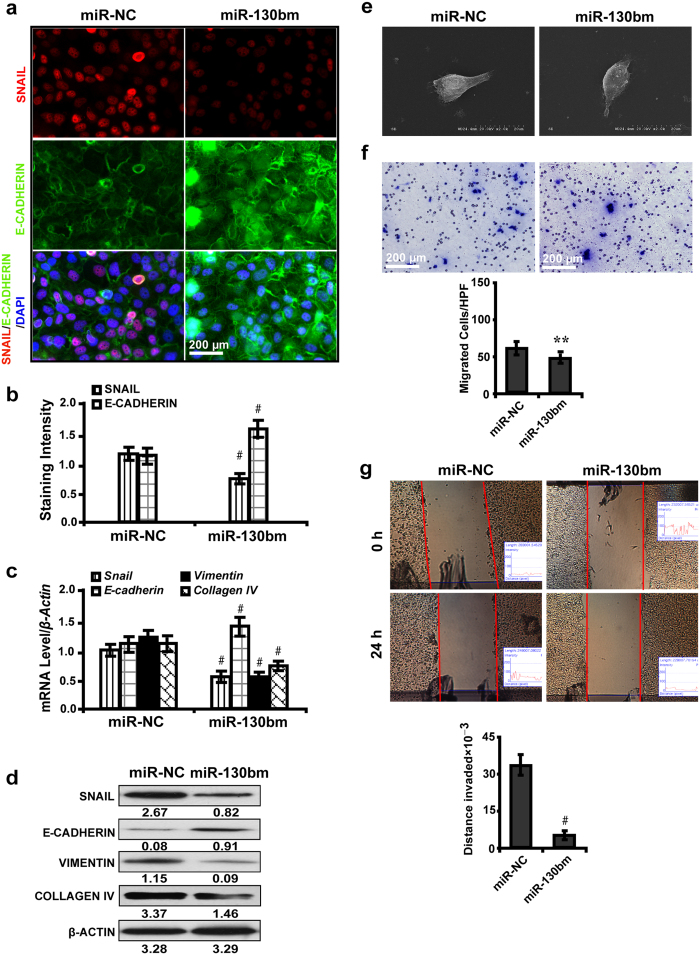
MiR-130b mimic suppresses *Snail*-induced EMT *in vitro*. (**a**) MiR-130b mimic decreased the expression of SNAIL but increased E-CADHERIN (immunofluorescence). (**b**) Decreased SNAIL but increased E-CADHERIN expression by quantification of the staining intensity. (**c**) MiR-130b mimic downregulated the mRNA level of *Snail*, *Vimentin* and *Collagen IV*, but upregulated *E-cadherin* by qRT-PCR. (**d**) MiR-130b mimic decreased SNAIL, VIMENTIN and COLLAGEN IV but increased E-CADHERIN by Western blot analysis. (**e**) NRK-52E cells retained the epithelial feature with miR-130b mimic treatment by SEM. (**f**) Decreased migrated cells with miR-130b mimic treatment by transwell assay. (**g**) Shorter invaded distance in NRK-52E cells treated with miR-130b mimic by wound healing assay. Results are presented as mean ± SD of three independent experiments. **P *<* 0.01; ^#^*P* *<* 0.0001. miR-NC, miRNA mimic negative control; miR-130bm: miR-130b mimic.

To further explore whether miR-130b overexpression could rescue the effect of *Snail*-induced EMT process, we therefore administered miR-130b mimic to high glucose cultured NRK-52E cells and detected the downstream gene expressions and biological features. Immunofluorescence microscopy revealed that miR-130b overexpression led to decreased expression of SNAIL but increased E-CADHERIN (Fig. 4a,b). MiR-130b mimic downregulated the mRNA level of *Snail*, *Vimentin* and *Collagen IV*, but upregulated *E-cadherin* as shown by quantitative real-time RT-PCR analysis (Fig. 4c). Western blot analysis demonstrated that miR-130b enrichment reduced the expression of SNAIL, VIMENTIN and COLLAGEN IV but increased E-CADHERIN (Fig. 4d). MiR-130b mimic did not cause phenotypic changes of NKR-52E cells (Fig. 4e) but inhibited the cells to migrate (Fig. 4f) and invade (Fig. 4g). These data clearly suggest a role of miR-130b in repressing the EMT process in high glucose cultured NRK-52E cells *in vitro*.

### High glucose inhibits miR-130b and regulates *Snail*-induced downstream gene expressions *in vitro*

**Figure 5:**
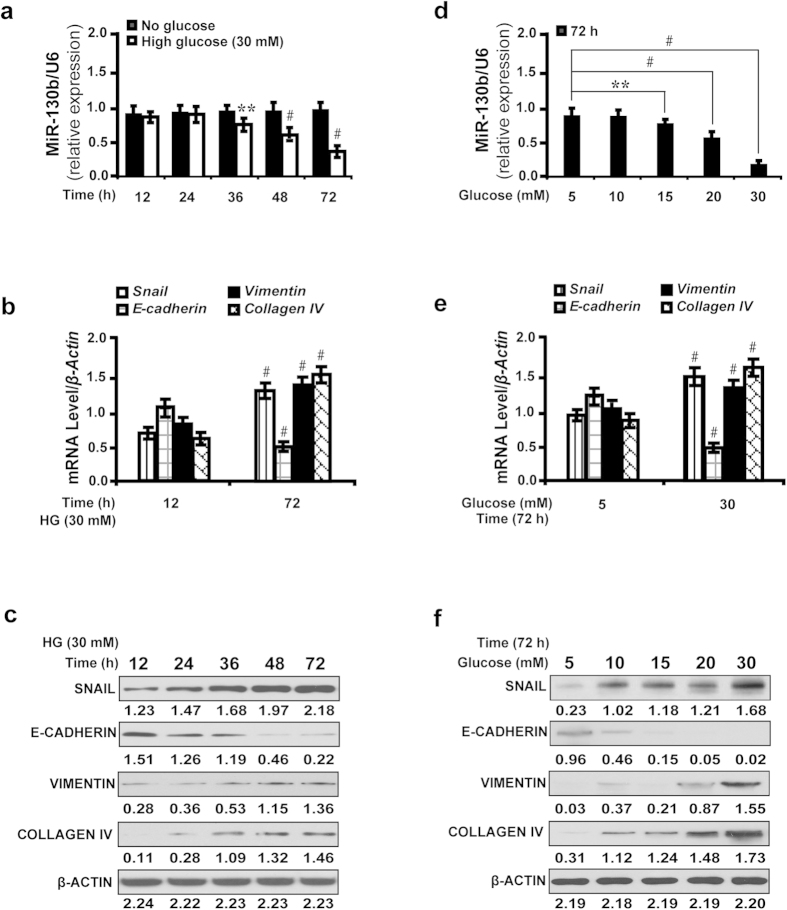
High glucose inhibits miR-130b and regulates *Snail*-induced downstream gene expressions *in vitro*. (**a**) High glucose (30 mM) reduced miR-130b expression in a time-dependent manner. (**b**) High glucose (30 mM) increased the level of *Snail*, *Vimentin*, and *Collagen IV*, but decreased *E-cadherin* by qRT-PCR and (**c**) Western blot analyses. (**d**) Increasing concentrations of glucose from 5 to 30 mM reduced miR-130b expression in a dose-dependent manner at 72 h. (**e**) Increased *Snail*, *Vimentin*, and *Collagen IV*, but decreased *E-cadherin* expression stimulated with increasing concentrations of glucose at 72 h by qRT-PCR and (**f**) Western blot analyses. Results are presented as mean ± SD of three independent experiments. *P *<* 0.05; **P *<* 0.01; ^#^*P* *<* 0.0001. HG, high glucose.

To investigate whether high glucose affects the miR-130b level, we treated NRK52E cells with increasing concentrations of glucose and detected the expression of miR-130b and downstream genes at different time points from 12 to 72 hours. We found high glucose reduced miR-130b expression level in a time dependent manner (Fig. 5a). Correspondingly, stimulation with high glucose (30 mM) increased the level of *Snail*, *Vimentin*, and *Collagen IV*, but decreased *E-cadherin* in a time dependent manner as shown by qRT-PCR (Fig. 5b) and Western blot analyses (Fig. 5c). Increasing concentrations of glucose reduced miR-130b expression in a dose dependent manner (Fig. 5d). The level of *Snail*, *Vimentin*, and *Collagen IV* increased, but *E-cadherin* decreased as shown by qRT-PCR (Fig. 5e) and Western blot analyses (Fig. 5f). To further investigate the effects of miR-130b inhibition or overexpression on downstream gene expressions in the presence of high glucose, we treated NRK52E cells with miR-130b inhibitor (miR-130bi) or miR-130b mimic (miR-130bm). Under high glucose microenvironment (see Supplementary Fig. S1 online), the level of miR-130b decreased or increased with miR-130b inhibitor or miR-130b mimic treatment, respectively. As demonstrated by qRT-PCR and Western blot analyses, miR-130b inhibitor upregulated *Snail*, *Vimentin*, and *Collagen IV*, but downregulated *E-cadherin*, however, miR-130 mimic had the opposite effect. These data indicate high glucose as a pivotal factor for the expression of miR-130b and regulating miR-130b expression affects *Snail*-induced downstream gene expressions *in vitro*.

### Requirement of *Snail* for the miR-130b antagonism effect on downstream gene expressions *in vitro*

**Figure 6:**
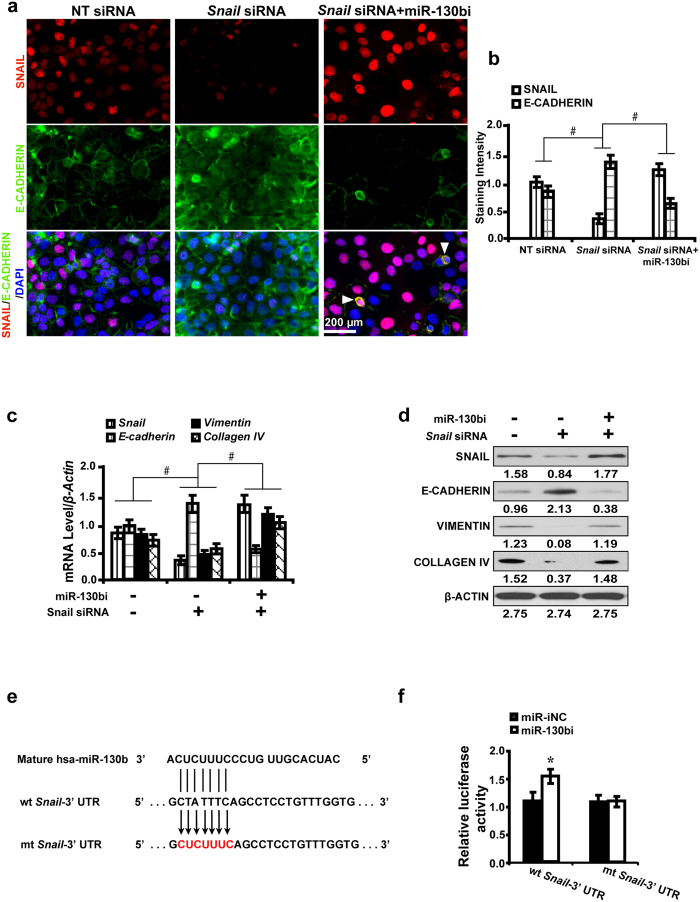
Requirement of *Snail* for the miR-130b antagonism effect on downstream gene expressions *in vitro*. (**a**) MiR-130bi restored SNAIL expression but further downregulated E-CADHERIN induced by *Snail* siRNA as shown by immunofluorescence and (**b**) quantification of the staining intensity. Arrowheads indicated co-localized SNAIL and E-CADHERIN. (**c**) MiR-130b inhibitor attenuated the silencing effect of *Snail* siRNA on the expression of *Snail*, *Vimentin* and *Collagen IV*, and downregulated *E-cadherin* by qRT-PCR and (**d**) Western blot analyses. (**e**) MiR-130b and its putative binding sequence in the 3′-UTR of *Snail*. The mutant *Snail* binding site was generated in the complementary site for the seed region of miR-130b. (**f**) MiR-130b inhibitor led to a noticeable increase in the luciferase activity of wt 3′-UTR of *Snail.* Results are presented as mean ± SD of three independent experiments. *P *<* 0.05; ^#^*P* *<* 0.0001. NT: non-targeting; siRNA: small interfering RNA; miR-iNC: miRNA inhibitor negative control; miR-130bi: miR-130b inhibitor; wt: wild type; mt: mutant type.

To explore whether the effect of miR-130b inhibition on downstream gene expressions depended on *Snail*, *Snail* siRNA was used to treat NRK52E cells following the treatment with miR-130b inhibitor. As shown by immunofluorescence microscopy (Fig. 6a) and quantification of the staining intensity (Fig. 6b), upon *Snail* silencing, SNAIL expression was significantly decreased with upregulated E-CADHERIN expression. However, when *Snail*-silenced cells were further treated with miR-130b inhibitor, the expression of SNAIL restored but E-CADHERIN considerably decreased. Furthermore, quantitative real-time RT-PCR (Fig. 6c) and Western blot analyses (Fig. 6d) revealed that miR-130b inhibitor obviously attenuated the silencing effect of *Snail* siRNA on the expression of Snail, Vimentin and Collagen IV and downregulated E-cadherin expression. To further demonstrate that *Snail* is directly targeted by miR-130b in NRK52E cells, we investigated whether miR-130b directly interacted with the 3′-UTR of *Snail* mRNA using a dual-luciferase reporter assay. MiR-130b inhibitor led to a noticeable increase in the luciferase activity of wild-type 3′-UTR of *Snail* but not the mutant (Fig. 6f). These results suggest that miR-130b directly suppresses *Snail* that subsequently regulates EMT and fibrosis related gene expressions.

### MiR-130b regulates the expression of EMT markers and renal tubulointerstitial fibrosis *in vivo*

**Figure 7:**
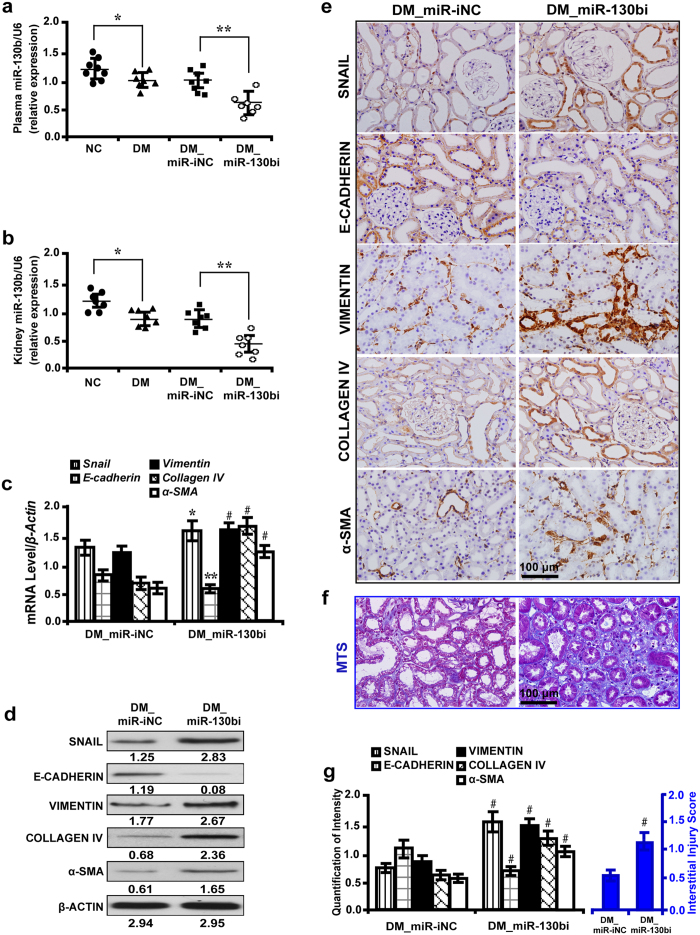
MiR-130b inhibitor increases EMT markers and promotes renal tubulointerstitial fibrosis *in vivo.* (**a**) Decreased miR-130b in plasma and (**b**) kidney samples of diabetic rats and miR-130b inhibitor further reduced its expression. (**c**) MiR-130b inhibitor increased *Snail*, *Vimentin*, *Collagen IV* and *α-SMA*, but decreased *E-cadherin* by qRT-PCR and (**d**) Western blot analysis. (**e**) MiR-130b inhibitor upregulated SNAIL, VIMENTIN, COLLAGEN IV and α-SMA, but decreased E-CADHERIN by immunohistochemistry. (**f**) Increased renal tubulointerstitial fibrosis in diabetic rats treated with miR-130b inhibitor by MTS. (**g**) Quantification for immunohistochemistry and (**h**) interstitial injury score. Results are presented as mean ± SD of three independent experiments. *P *<* 0.05; **P *<* 0.01; ^#^*P* *<* 0.0001. NC, normal control; DM, diabete mellitus; DM_miR-iNC, diabetic rats treated with miRNA inhibitor negative control; DM_miR-130bi, diabetic rats treated with miR-130b inhibitor; MTS, Masson’ s trichrome stain.

**Figure 8:**
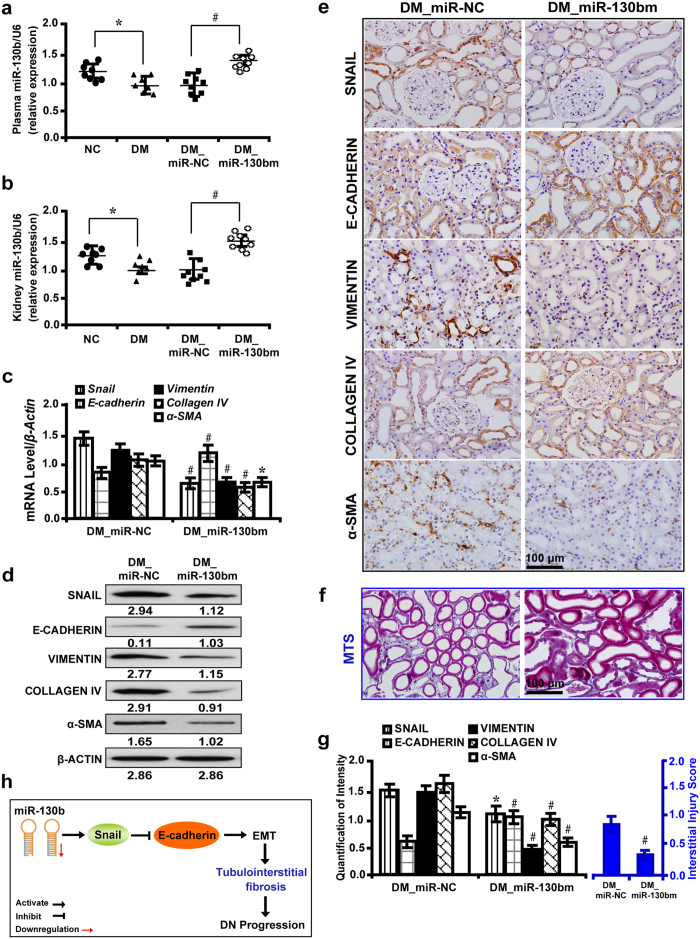
MiR-130b mimic decreases the expression of EMT markers and inhibits renal tubulointerstitial fibrosis *in vivo.* (**a**) Decreased miR-130b in plasma and (**b**) kidney samples of diabetic rats and miR-130b mimic upregulated its expression. (**c**) MiR-130b mimic decreased *Snail*, *Vimentin*, *Collagen IV* and *α-SMA*, but increased *E-cadherin* by qRT-PCR and (**d**) Western blot analysis. (**e**) MiR-130b mimic downregulated the level of SNAIL, VIMENTIN, COLLAGEN IV and α-SMA, but increased E-CADHERIN by immunohistochemistry. (**f**) Improved renal tubulointerstitial fibrosis in diabetic rats treated with miR-130b mimic by MTS. (**g**) Quantification of the staining intensity for immunohistochemistry (left panel) and interstitial injury score (right panel). (**h**) A hypothetical model illustrated that miR-130b regulates renal tubulointerstitial fibrosis through *Snail/Ecadherin*-mediated EMT process in diabetic nephropathy. Results are presented as mean ± SD of three independent experiments. *P *<* 0.05; #*P* *<* 0.0001. NC, normal control; DM, diabete mellitus; DM_miR-NC, diabetic rats treated with miRNA mimic negative control; DM_miR-130bm, diabetic rats treated with miR-130b mimic; MTS, Masson’ s trichrome stain.

MiR-130b in plasma and tissue samples were decreased in diabetic rats and further depleted with miR-130b inhibitor treatment (Fig. 7a,b). MiR-130b inhibitor caused considerable increase in the level of BUN, serum creatinine, β2-microglobulin and albuminuria (see Supplementary Table S1 online), but miR-130b mimic had the opposite effects (see Supplementary Table S2 online). Correspondingly, miR-130b inhibition elevated the level of *Snail*, *Vimentin*, *Collagen IV* and *α-SMA*, but decreased *E-cadherin* as detected by qRT-PCR (Fig. 7c) and Western blot analyses (Fig. 7d). Immunohistochemistry (Fig. 7e) and quantification of the staining intensity (Fig. 7g, left panel) revealed that miR-130b inhibition upregulated the expression level of SNAIL, VIMENTIN, COLLAGEN IV and α-SMA, but decreased E-CADHERIN. MTS (Fig. 7f) and interstitial injury score (Fig. 7g, right panel) demonstrated that miR-130b inhibitor increased renal tubulointerstitial fibrosis. Conversely, miR-130b overexpression had the opposite effects (Fig. 8). Furthermore, plasma miR-130b level was negatively associated with albuminuria in diabetic rats treated with miR-130b inhibitor or mimic (see Supplementary Fig. S2 online).

## Discussion

Our findings indicate a critical role of miR-130b in regulating renal tubulointerstitial fibrosis in diabetic nephropathy. We demonstrated that plasma miR-130b was decreased in DN patients and correlated with declined renal function. We also found that miR-130b level was decreased in high glucose cultured NRK-52E cells, plasma or kidney tissue samples of streptozotocin-induced diabetic rats. MiR-130b targeted *Snail* and miR-130b abrogation enhanced the expression of markers related to EMT and fibrosis. Moreover, *Snail* interacted and inversely correlated with *E-cadherin*, regulating downstream gene expressions. Taken together, these data implicate miR-130b as a valuable biomarker in evaluating the severity of renal tubulointerstitial fibrosis in diabetic nephropathy.

The present study also revealed that in renal biopsies of DN patients, tubular expression of SNAIL, VIMENTIN, COLLAGEN IV and α-SMA increased but E-CADHERIN decreased, corresponded with increased tubulointerstitial fibrosis. In high glucose cultured NRK-52E cells, miR-130b depletion upregulated *Snail* expression and induced phenotypic transformation of the cells from sessile epithelial to invasive mesenchymal state, with increased ability to migrate and invade. Moreover, miR-130b inhibitor increased renal tubulointerstitial fibrosis in diabetic rats. Conversely, administration of miR-130b mimic to high glucose cultured NRK-52E cells and diabetic rats remarkably decreased the expression of *Snail* and attenuated renal fibrosis. These results suggest that *Snail*-induced EMT process is the pivotal mechanism in triggering a cascade of reactions leading to fibrosis.

Altered microRNA expression and perturbed signaling pathways have been associated with epithelial to mesenchymal transition. Here, we found that miR-130b regulated the expression of *Snail* and downstream gene expressions associated with renal fibrosis. Furthermore, we indentified the co-localization and inverse correlation between SNAIL and E-CADHERIN in renal biopsy samples of DN patients, further indicating that *Snail* is a critical regulator in mediating the EMT process and tubulointerstitial fibrosis in diabetic nephropathy. This is in agreement with previous studies showing that *Snail* is one of the genes, which repressed E-CADHERIN expression and promoted EMT in hepatocytes^19^.

In diabetes, renal tubular EMT is an important event in initiating tubulointerstitial fibrosis and contributes to the disease progression and declined renal function^20^,^21^. Emerging evidences indicate that EMT can be provoked by a variety of stimuli that trigger specific intracellular signalling pathways, such as TGF-β^22^,^23^. Wnt^24^ and Smad2/3 signaling^25^. The common feature of these pathways is that they regulate the activity of *E-cadherin*, the main molecule mediating the EMT process. As a typical epithelial phenotypic marker, *E-cadherin* is an important molecule in the maintenance of the epithelial phenotype; the reduction and loss of its expression have been reported to result in a decline in cell adhesion. *Snail* transcription factor, an initiator of EMT, has been demonstrated to interact directly with its putative binding sites in the *E-cadherin* promoter and hence inhibit the expression of E-CADHERIN protein^26^,^27^,^28^. Snail expression is regulated at the transcriptional level by various signaling molecules and some post-translational modifications influence Snail stability, subcellular localization and activity^29^,^30^. Recent evidence indicates that Snail plays a pivotal role in tubulointerstitial fibrosis in patients with chronic kidney disease and progressive nephropathy^31^ and exerts its function through repressing E-cadherin expression in several kinds of diseases^32^. Snail has also been shown to induce the expression of α-SMA, fibronectin, collagen I, genes directly implicated in myofibroblast activation and interstitial matrix production^4^. The present study highlights the notion that *Snail*-induced suppression of *E-cadherin* activity contributes to miR-130b-mediated tubulointerstitial fibrosis in diabetic nephropathy. However, we did not investigate these associations in other forms of kidney diseases, which needs further investigation in future studies.

The impact of EMT in renal fibrosis is still controversial. Since the first description of the cellular evidence for EMT by Greenburg and Hay^33^ in the early 1980s, there have been numerous studies supporting the notion that the key lineage effector cells mediating EMT process and renal fibrosis include interstitial fibroblasts^34^, pericytes^35^, local mesenchymal stem cells^36^ or the injured epithelial cells^37^. However, this theory has been debated by a small number of studies^35^,^38^ demonstrating that the EMT model may not be translated to the *in vivo* scenario and myofibroblasts derive from interstitial pericytes/perivascular fibroblasts instead of renal tubular epithelial cells. On the contrary, one recent study using genetically modified approaches has revealed that EMT was visible and quantifiable in the fibrotic kidney sections of four renal disease models *in vivo*^39^. Such disparities may have arisen from varying experimental conditions such as different disease models, the mouse strain, and type of genetic alteration used.

The current view of EMT in renal fibrosis centers on the inflammatory stimuli which induces conserved signaling pathways in epithelia that regulate EMT in embryonic epithelia^40^. Under this state, adult tubular epithelial cells respond by acquisition of a mesenchymal phenotype leading to formation of a fibroblast^41^. In fact, the exact percentage of tubular epithelial cells that become fibroblasts in a diseased kidney is entirely unknown and the transition does not have to go that far to induce renal fibrosis. Apparently, real time monitoring of EMT *in vivo* would be the best approach to view epithelial cells transitioning into fibroblasts and migrating to the interstitium. Unfortunately, this is not feasible with current technology. Most studies use co-immunostaining for markers of epithelial cell lineage, fibroblast markers, and activation of the EMT transcriptional program, particularly nuclear translocation of Snail^41^, in which Snail signaling is active and E-cadherin is lost^42^. The nuclear to cytoplasmic translocation of Snail and co-localization with E-cadherin in both NRK52E cells and renal biopsies of DN patients indicate the involvement of EMT in the present study. Additionally, the findings of phenotypic changes in cultured cells and the expression changes of molecules related with EMT and fibrosis, such as E-cadherin, Vimentin, and Collagen IV support the notion that the EMT process exists in the present model. Future studies warrant the exploration of renal tubular epithelial cells transitioning into fibroblasts in real time when appropriate technology is available.

Studies by Paula Fioretto *et al.* have shown that in human the kidney lesions in type 1 and type 2 are similar^43^. In the present study, we used 65 mg/kg of streptozotocin to induce type 1 diabetes. We monitored the renal interstitial changes and expression levels of miR-130b, Snail and related molecules at four, eight and 12 weeks. Differences began to appear at four weeks and reached statistical significance at 12 weeks. The morphology of the diabetic rat kidneys at 12 weeks resembled diabetic nephropathy of type 2 diabetes, including glomerular hypertrophy, mesangial expansion, and especially renal interstitial fibrosis. Since the focus of our present study is to analyze the interstitial fibrosis of diabetic nephropathy, the morphological changes of streptozotocin-induced diabetic rats at 12 weeks met our experimental needs.

The present study has some limitations that may impact on the interpretation of the data. 1) The relatively small size of effective plasma miRNAs extracted for analysis, which merits validations in expanded samples. 2) Since alterations in the levels of plasma miRNAs as biomarkers do not necessarily reflect the biological functions of miRNAs expression inside cells, the roles and significance of miRNAs deregulated in plasma samples from patients with diabetic nephropathy still warrant determination. Nevertheless, the current study provides promising evidences for future analysis using plasma miRNAs to evaluate the pathophysiology of diabetic nephropathy. Plasma miR-130b can be extrapolated to quantifying the severity of renal tubulointerstitial fibrosis in diabetic nephropathy.

In conclusion, this study provides a novel mechanism that miR-130b attenuates tubulointerstitial fibrosis in diabetic nephropathy through repression of *Snail*-induced EMT. Targeting miR-103b might be an alternative approach to suppress renal tubulointerstitial fibrosis in diabetic nephropathy.

## Methods

All methods were carried out in accordance with the approved guidelines.

### Patients and Specimens

We started with 152 diabetic patients, including 27 from the Division of Nephrology in Nanfang Hospital and 125 from the Department of Renal Pathology in King Medical Diagnostics Center in Guangzhou from 2012 to 2014. The inclusion criteria were: 1) type 2 diabetic patients; 2) the indications for performing the renal biopsy were proteinura, microscopic hematuria, and fast drop in renal function. The exclusion criteria were: 1) patients who have taken toxic or herbal medicine which may have caused kidney disease; 2) a second disease was found on the examination of kidney biopsy. Sixty-six patients were excluded and a total of 86 type 2 diabetic patients undergoing renal biopsy were selected for analysis (17 from the Division of Nephrology in Nanfang Hospital and 69 from the Department of Renal Pathology in King Medical Diagnostics Center). For each of the two cohorts, the biopsy morphologic findings include tubular atrophy and interstitial fibrosis, diabetic glomerulosclerosis (hypertrophy, thickened basement membranes, mesangial matrix accumulation and nodules), and arteriosclerosis. The medications these patients have been receiving mainly comprise of biguanides, angiotensin-converting enzyme inhibitors, angiotensin receptor blockers, calcium channel blockers and hydroxymethylglutaryl-CoA Reductase Inhibitors.

Peripheral blood samples from 27 out of the 86 DN patients and 20 healthy controls were collected and plasma was obtained by standard protocol. Total RNA was extracted from the blood using Trizol Reagent (Invitrogen, Carlsbad, CA, USA). To extract miRNAs, one synthetic oligonucleotide corresponding to miRNAs non-existent in humans (3′-UUUCAUGUCGAUUUCAUUUCAUG-5′) was spiked-in for quality control before RNA isolation according to the manufacturer’s instructions. To validate the success of each extraction, the thermal cycle (Ct) values obtained for a serial dilution of these miRNAs were assessed. Blood glucose concentrations were evaluated using a glucose analyzer (Accu-check Advantage; Roche, Mississauga, ON, Canada). Serum β2-microglobulin (β2-MG) was examined using an enzyme-linked immunosorbent assay (ELISA) kit (Roche Diagnostics GmbH, Mannheim, Germany). A Beckman Coulter AU480 Chemistry Analyzer (Beckman, CA, USA) was used to determine the level of blood urea nitrogen (BUN), serum creatinine and urine albumin. All experiments were repeated in triplicate.

Total 86 formalin-fixed paraffin-embedded renal biopsy tissue samples were used for immunohistochemical analyses and 42 available snap-frozen samples from among the 86 cases were selected for immunofluorescence co-labeling. Written informed consent was obtained from each patient and tissue specimens were processed according to the protocols of the Ethnics Committee from Southern Medical University and King Medical Diagnostics Center. In all specimens, the morphological diagnosis of DN was confirmed by two individual renal pathologists (JG and XB). Normal human renal tissues (n = 20) from distant portions of kidney tumor were used as controls.

### Cell Culture and Studies

Rat kidney tubular epithelial cells (NRK52E; American Type Culture Collection, Rockville, MD, USA) were maintained in Dulbecco’s Modified Eagle’s medium (DMEM)/F-12 containing 10% fetal bovine serum, penicillin (200 U/ml), and streptomycin (200 μg/ml) (Gibco BRL, Grand Island, NY, USA). Cells were cultured in high glucose (30 mM) for one week, grown to 80%–90% confluence and made quiescent by incubation overnight in a serum-free medium.

### Transfection of miRNA mimic, inhibitors and small interfering RNA

MiR-130b inhibitor (miR-130bi), miR-130b mimic (miR-130bm), or the appropriate negative controls (NC) of miRNA inhibitor (miR-iNC) and miRNA mimic (miR-NC), respectively, were purchased from GenePharma (Shanghai, China) and transfected at a final concentration of 50–100 nM in the cells using HiPerFect Transfection Reagent (Qiagen, Hilden) according to the manufacturer’s recommendations. Expression of murine Snail was knocked down with small interfering RNA (siRNA) duplexes using Oligofectamine (Invitrogen, Carlsbad, CA).

### Luciferase reporter assay

The predicted 3′-untranslated regions (UTR) sequence of *Snail* interacting with miR-130b and mutated sequences within the predicted target sites were synthesized and inserted into the pRL-TK control vector (Promega, Madison, WI, USA). NRK52E cells transfected with 120 ng miR-130b inhibitor or negative controls, followed by co-transfection with 30 ng of the wild-type or mutant 3′-UTR of *Snail* using 0.45 μL of Fugene (Promega, Madison, WI, USA). Luciferase assay was carried out on extracts from the cells 48 hours post transfection and measured using Dual-Luciferase Assay System (Promega, Madison, WI, USA). pRL-TK expressing Renilla luciferase was co-transfected as an internal control. Data were normalized by the ratio of Firefly and Renilla luciferase activities.

### Scanning electron microscopy

Cells cultured on coverslips were fixed with 2.5% glutaraldehyde overnight at 4 °C, followed by washing in phosphate-buffered saline (PBS, pH = 7.4) and dehydration. Hitachi S-3000N Scanning Electron Microscope (SEM; Hitachi, Tokyo, Japan) was used to examine the cells and images were taken at the magnification of ×2000.

### Migration assay

Cells (1.0 × 10^6^ cells/ml) in serum-free medium were added to the top chamber of 24-well transwell plates (8 mm pore size; Corning Star, Cambridge, MA, USA) and 600 μl of complete medium with 10% FBS into the bottom chamber. The assembled chamber was incubated at 37 °C in a humidified, 5% CO_2_ cell culture incubator for 24 hours and fixed with 10% formalin and stained with crystal violet for visualization.

### Wound healing assay

Cells (2.0 × 10^5^ cells/well) were plated in 6-well plates and grown to 80% confluence. The individual wells were wounded by scratching with a pipette tip and incubated with medium containing no FBS to various time points. Wells were photographed under phase-contrast microscopy (×10).

### Animal Studies

Male Sprague Dawley rats (6–*8 weeks of age) were kept in the Animal Center of Nanfang Hospital according to the policy of the Committee for Animal Usage. Diabetes was induced with streptozotocin (65 mg/kg; S0130; Sigma-Aldrich, MO, USA) according to the protocol described previously*^44^,^45^*. To investigate the effect of miR-130b inhibition or overexpression on renal tubulointerstitial fibrosis, miR-130b inhibitor (4 ng/mm*^*3*^*) or miR-130b mimic (2 ng/mm*^*3*^) was injected peritoneally to diabetic rats every other day. The appropriate negative control miRNAs, indicated as miRNA inhibitor (miR-iNC) and miRNA mimic (miR-NC), respectively, were included as controls.

### Groups

Four groups (9–10 per group) were studied: diabetic rats treated with miR-130b inhibitor (4 ng/mm^3^) (DM_miR-130bi), negative control miRNA inhibitor (DM_miR-iNC), miR-130b mimic (2 ng/mm^3^) (DM_miR-130bm), and negative control miRNA mimic (DM_miR-NC). No adverse or toxic effects were observed. Blood glucose level was measured every two weeks. The treatment continued until the renal tubulointerstitial fibrosis was present and the rats were euthanized at twelve weeks. The study protocols were approved by the Animal Ethics Committee at Nanfang Hospital, Southern Medical University, Guangzhou, China.

### Sample collection

Twelve weeks after the streptozotocin injection, blood was drawn from the tail vein and plasma samples were prepared for analyzing BUN, creatinine, β2-microglobulin and glucose level. Urine was collected for determination of albuminuria. Plasma miRNAs was extracted as described above and used for evaluation of miR-130b level. At 12 weeks after the induction of diabetes, rats were anesthetized with pentobarbital sodium (P3761, 30 mg/kg, Sigma-Aldrich, St. Louis, MO, USA) and kidney/body weight was obtained. Left kidneys were obtained and fixed in 10% formalin in PBS for 24 hours and embedded in paraffin for histological analysis. The right kidney was snap-frozen and stored at –80 °C for further analysis.

### Laser capture microdissection (LCM)

Frozen kidney tissues from normal control and diabetic rat were cut at 8 μm thickness and renal tubules and interstitium were microdissected using the PALM MicroBeam LCM system (Zeiss, Germany) according to previously published procedures^44^.

### Quantitative Real-time Reverse Transcription-PCR

Total RNA from NRK-52E cells and microdissected renal tubules were extracted using TRIzol reagent (MRC, Cincinnati, OH, USA). First strand cDNA was synthesized using 2 μg of total RNA treated with Moloney murine leukemia virus reverse transcriptase (Promega, Madison, WI, USA) according to the manufacturer’s instructions. Quantitative real-time reverse transcription-PCR (RT-PCR) analysis was performed in triplicate with Power PCR SYBR Green Master Mix (Applied Biosystems, Carlsbad, CA, USA) using the ABI PRISM 7500 FAST Real-TIME PCR System (Applied Biosystems) with results normalized to β-actin expression. The ΔΔCT method was used to calculate relative expression. Primer sequences used in RT-PCR are shown in Supplementary Table S3 online.

For miRNA expression analysis of the cells, human plasma samples and rat kidney tissues or plasma samples, RNA was reverse transcribed using miRScript PCR System and analyzed by qRT-PCR with the miScript SYBR Green PCR Kit using the specific miR-130b miScript Primer Assays (Qiagen, Hilden, Germany) according to the manufacturers’ instructions. Expression levels were normalized to the average of U6-snRNA. MiR-130b levels were calculated as fold change (2^–ΔΔCT^) with respect to normal controls. The mean value of miR-130b expression in glucose-free cultured cells was used as the calibrator. Target-specific reverse transcription and Taqman microRNA assays were performed using the Hairpin-it^TM^ miRNA qPCR Quantitation Kit (GenePharma, Suzhou) according to the protocol. The reactions were performed using the ABI PRISM 7500 FAST Real-TIME PCR System (Applied Biosystems, Carlsbad, CA, USA) with results normalized to snRNA U6 expression. The 2^−ΔΔCt^ method was used to calculate the relative expression. All experiments were performed in triplicate.

### Western Blot Analysis

Lysates from the cells and microdissected renal tubules from each experimental group were separated in parallel on two 10% denaturing sodium dodecyl sulfate-polyacrylamide gels, transferred onto nitrocellulose membranes, blocked with 5% nonfat milk in 0.1% tris buffered saline with Tween-20 (TBST), and probed using antibodies to rabbit polyclonal anti-*S*NAIL antibody (1:100, ab180714, Abcam, Cambridge, UK), mouse anti-*E-*CADHERIN antibody antibody (1:100, ab76055, Abcam, Cambridge, UK), mouse monoclonal VIMENTIN (D21H3) antibody (1:100, #5741, cell signaling technology, MA, USA), and rabbit polyclonal anti-*C*OLLAGEN IV antibody (1:100, ab6586, Abcam, Cambridge, UK) at 4 °C overnight. After washing, the secondary antibody (horseradish peroxidase-labeled IgG anti-rabbit/mouse antibody, Invitrogen, USA) was used at 1:3000 dilution for 1 hour at room temperature. The supersignal-enhanced chemoluminescent substrate (Pierce Biotechnology, Inc., Rockford, IL, USA) was applied to the probed membrane and exposed for 10 minutes before the protein bands were visualized on radiograph films (Super Rx, Fuji Photo Film, Tokyo, Japan).

### Immunofluorescence and Immunohistochemical Analysis

NRK52E cells, tissue samples from the patients and rats were labeled with antibodies to SNAIL (1:100), E-CADHERIN (1:100), VIMENTIN (1:100), COLLAGEN IV (1:100) and α-smooth muscle actin (α-SMA) (1:300). For immunofluorescence staining, Alexa Fluor 594-conjugated goat anti-mouse IgG and Alexa Fluor 488-conjugated goat anti-rabbit IgG (1:1000, Invitrogen, Cambridge, MA, USA) were used for secondary antibodies, nuclei were counterstained with 4,6-diamidino-2-phenylindole (DAPI, Sigma-Aldrich, St. Louis, MO, USA) and coverslipped with aqueous mounting medium (CTS011, BD Bioscience, MN, USA). For immunohistochemistry, EnVision™ Detection Systems Peroxidase/diaminobenzidine (DAB), Rabbit/Mouse kit (K4065, Dako, Carpinteria, CA, USA) was used. Nuclei were counterstained with hematoxylin and coverslipped with Permount mounting medium (00-4960-56, eBioscience, CA, USA).

Samples were evaluated semiquantitatively by systematically selecting without bias twenty fields for analysis. Images were taken with a BX51 light microscope (Olympus, Tokyo) with appropriate filters. Staining intensity was measured using Image J analysis software (Image J 1.44, National Institute of Health, USA). PBS instead of primary antibodies served as a negative control.

### Evaluation of Renal Tubulointerstitial Fibrosis

Five-μm thick paraffin sections were cut for Masson’s trichrome stain (MTS). Area of tubulointerstitial fibrosis was measured using the Image J analysis software by evaluating areas of the injured tubules and interstitium and recorded as interstitial injury score^46^.

### Statistical Analysis

Data are presented as mean ± SD. Mann-Whitney U-test (for non-parametric data) or paired student’s t-test (for parametric data) were used to test statistical significance. Pearson correlation analysis was used to analyze correlations between plasma miR-130b and biological parameters, and between SNAIL and E-CADHERIN expression. All statistical tests were performed using SPSS 12.0 (SPSS, Inc., Chicago, IL, USA). The significance level is set at 0.05 to indicate statistical significance (□*P < 0.05; **P < 0.01; #*P* < 0.0001).

## Additional Information

**How to cite this article**: Bai, X. *et al.* MicroRNA-130b improves renal tubulointerstitial fibrosis via repression of Snail-induced epithelial-mesenchymal transition in diabetic nephropathy. *Sci. Rep.* **6**, 20475; doi: 10.1038/srep20475 (2016).

## Supplementary Material

Supplementary Information
