# Supplementary material for: MicroRNA-130b improves renal tubulointerstitial fibrosis via repression of Snail-induced epithelial-mesenchymal transition in diabetic nephropathy
Source: Sci Rep. 2016 Feb 3;6:20475. doi: 10.1038/srep20475 (PMC4738324; doi:10.1038/srep20475)

SUPPLEMENTARY INFORMATION

MicroRNA-130b improves renal tubulointerstitial fibrosis via repression of Snail-induced epithelial-mesenchymal transition in diabetic nephropathy

Xiaoyan Bai[1, §, *], Jian Geng[2, 3, §], Zhanmei Zhou[1, §], Jianwei Tian[1, §], Xiao Li[4]

[1]Division of Nephrology, Nanfang Hospital, Southern Medical University, National Clinical Medical Research Center for Kidney Disease, National Key Lab for Organ Failure Research, Guangdong Provincial Institute of Nephrology, Guangzhou, 510515, China

[2]Department of Pathology, Nanfang Hospital, Southern Medical University, Guangzhou, 510515, China

[3]Department of Renal Pathology, King Medical Diagnostics Center, Guangzhou, 510330, China

[4]Department of Emergency, Nanfang Hospital, Southern Medical University, Guangzhou, 510515, China

*Correspondence and requests for materials should be addressed to X.B. (e-mails: xiaoyanb@126.com or xiaoyanbai@smu.edu.cn)

[§]these authors contributed equally to this study

i. Supplementary Tables

Supplementary Table S1. Biological parameters for diabetic rats treated with miR-130b inhibitor at week 12

**Variables**	**DM_miR-iNC (n=10)**	**DM_miR-130bi (n=9)**
Serum blood urine nitrogen (mmol/L)	7.95±1.06	9.28±0.15**
Serum creatinine (umol/L)	105.67±7.28	112.33±1.97***
Serum β2-microglobulin (mcg/mL)	0.09±0.11	0.15±0.08 #
Albuminuria (μg/24h)	1.26±0.04	1.46±0.10#
Blood glucose (mmol/L)	24.57±2.06	24.09±0.13
Body weight (g)	184.92±2.18	185.24±2.16
Kidney weight (g)	1.85±1.03	1.85±1.06
Kidney/body weight (%)	1.01	1.00

DM_miR-iNC, diabetic rats treated with miRNA inhibitor negative control; DM_miR-130bi, diabetic rats treated with miR-130b inhibitor;

***P*<0.01; ****P*<0.001; #*P*<0.0001.

Supplementary Table S2. Biological parameters for diabetic rats treated with miR-130b mimic at week 12

**Variables**	**DM_miR-NC (n=10)**	**DM_miR-130bm (n=9)**
Serum blood urine nitrogen (mmol/L)	7.26±1.24	6.37±1.03**
Serum creatinine (umol/L)	112.4±2.38	101.5±2.37 *
Serum β2-microglobulin (mcg/mL)	0.12±0.06	0.07±0.01 **
Albuminuria (µg/24h)	1.27±0.03	0.84±0.06#
Blood glucose (mmol/L)	22.7±1.26	22.5±0.85
Body weight (g)	185.6±1.22	186.16±2.34
Kidney weight (g)	1.82±0.26	1.81±0.38
Kidney/body weight (%)	1.05	0.85

DM_miR-NC, diabetic rats treated with miRNA mimic negative control; DM_miR-130bm, diabetic rats treated with miR-130b mimic;

***P*<0.01; ****P*<0.001; #*P*<0.0001.

Supplementary Table S3. Primer sets used in real time RT-PCR

Genes (rat)	Forward Primer	Reverse Primer
Snail	5’-TCGCGAGCAGAGTTGTCTAC-3’	5’-TGCAGCTCGCTATAGTTGGG-3’
E-cadherin	5’-CCACCAGATGACGATACCCG -3’	5’-GCTTCAGAACCACTCCCCTC-3’
Vimentin	5’-TGAGATCGCCACCTACAGGA-3’	5’-GAGTGGGTGTCAACCAGAGG-3’
Collagen IV	5’-CCAAGGGAACCAGAGGCTTT-3’	5’-GTGCATCATAACATTTTACTGGACC-3’
α-SMA	5’- GAGGCACCACTGAACCCTAA-3’	5’- CATCTCCAGAGTCCAGCACA-3’
β-actin	5'- ATGATGATATCGCCGCGCTC -3'	5'- TCGATGGGGTACTTCAGGGT -3'

ii. Supplementary Figure Legend

Supplementary Figure S1. Effect of miR-130b inhibition or overexpression on downstream gene expression under high glucose microenvironment. (**A**) The level of miR-130b decreased or increased with miR-130b inhibitor or miR-130b mimic treatment, respectively. (**b**) MiR-130b inhibitor upregulated *Snail*, *Vimentin*, and *Collagen IV*, but downregulated *E-cadherin* by RT-PCR. (**c**) MiR-130b mimic downregulated *Snail*, *Vimentin*, and *Collagen IV*, but upregulated *E-cadherin* by RT-PCR. (**d**) MiR-130b inhibitor upregulated SNAIL, VIMENTIN, AND COLLAGEN IV, but downregulated E-CADHERIN, however, miR-130 mimic had the opposite effect by Western blot analysis. Results are presented as mean ± SD of three independent experiments. ∗P<0.05; ∗∗P<0.01; #P<0.0001. HG, high glucose; miR-130bi, miR-130b inhibitor; miR-130bm, miR-130b mimic.

Supplementary Figure S2. Inverse correlation between plasma miR-130b and albuminuria in diabetic rats. (**A**) Diabetic rats treated with DM_miR-iNC. (**b**) Diabetic rats treated with DM_miR-130bi. (**c**) Diabetic rats treated with DM_miR-NC. (**d**) Diabetic rats treated with DM_miR-130bm. ∗P<0.05; ∗∗P<0.01; #P<0.0001. DM_miR-iNC, diabetic rats treated with miRNA inhibitor negative control; DM_miR-130bi, diabetic rats treated with miR-130b inhibitor; DM_miR-NC, diabetic rats treated with miRNA mimic negative control; DM_miR-130bm, diabetic rats treated with miR-130b mimic.

Supplementary Figure S1

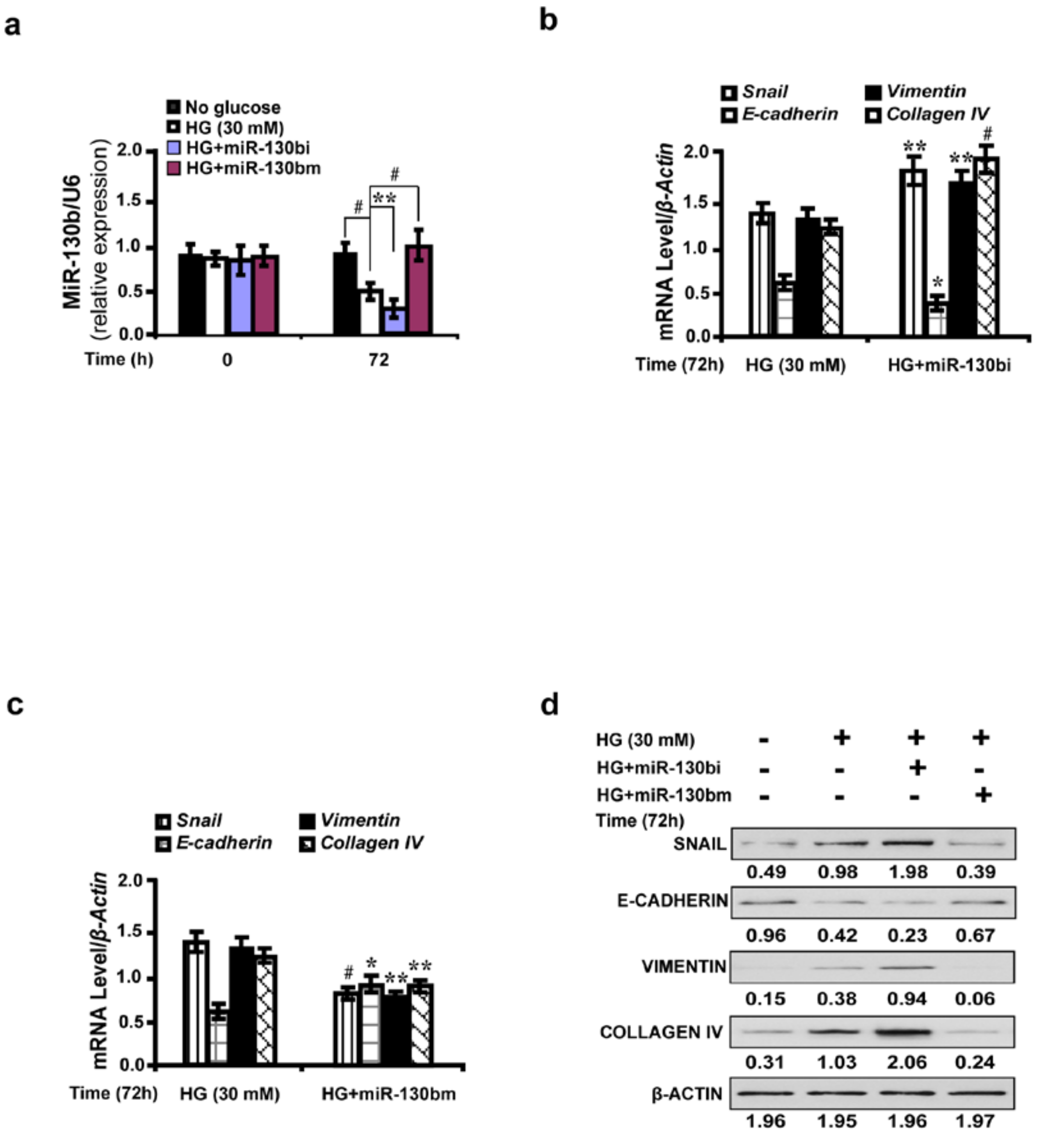

Supplementary Figure S2

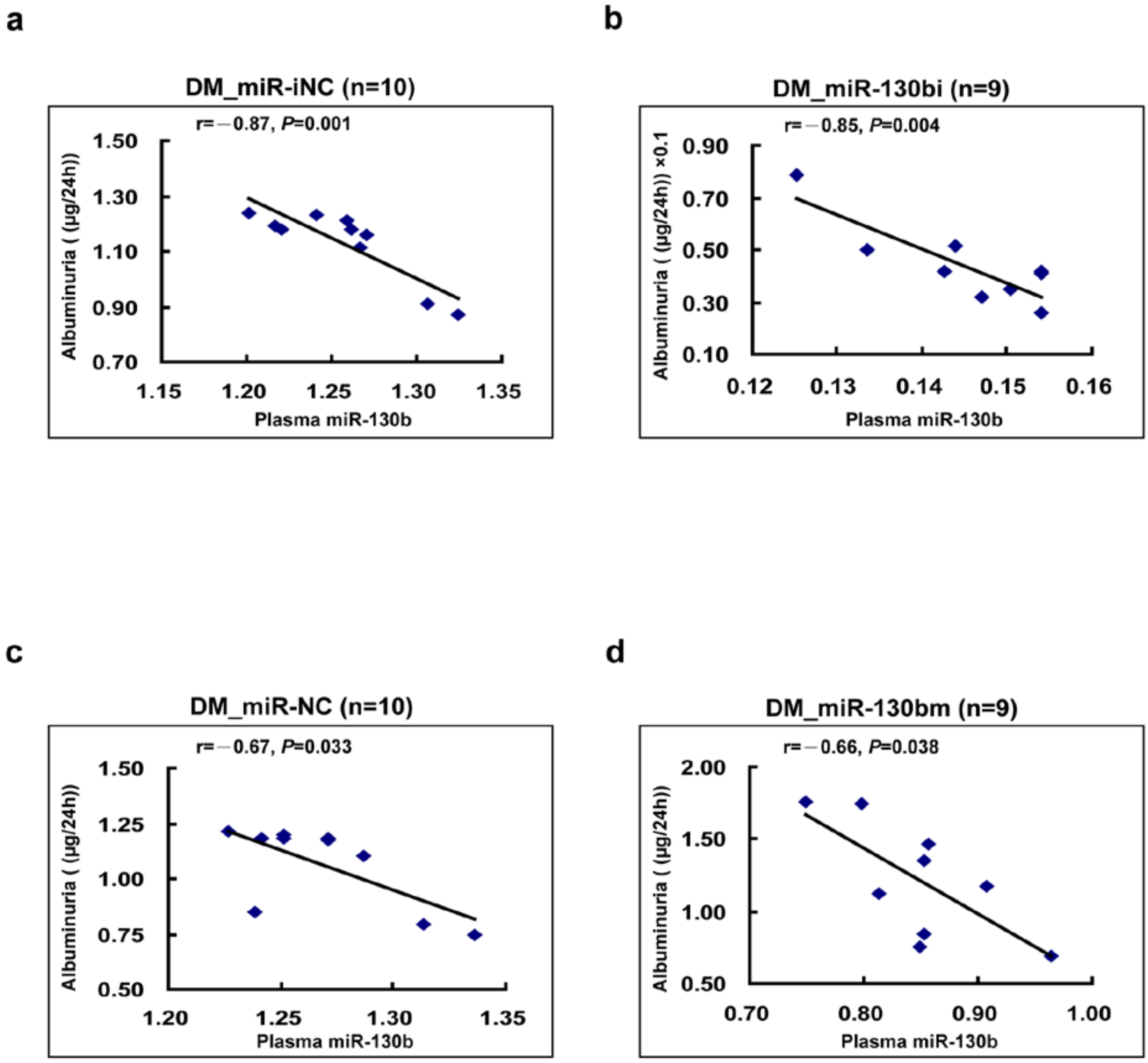

Supplement: Supplementary Information [file srep20475-s1.pdf]
